# Comparative Genomic Analysis of Transgenic Poplar Dwarf Mutant Reveals Numerous Differentially Expressed Genes Involved in Energy Flow

**DOI:** 10.3390/ijms150915603

**Published:** 2014-09-03

**Authors:** Su Chen, Shuang Bai, Guifeng Liu, Huiyu Li, Jing Jiang

**Affiliations:** State Key Laboratory of Tree Genetics and Breeding (Northeast Forestry University), 26 Hexing Road, Harbin 150040, China; E-Mails: suc@mtu.edu (S.C.); baishuang800826@163.com (S.B.); liuguifeng@126.com (G.L.); lihuiyu0519@163.com (H.L.)

**Keywords:** comparative genomic analysis, dwarf, photosynthesis, poplar

## Abstract

In our previous research, the *Tamarix androssowii* *LEA* gene (*Tamarix androssowii* late embryogenesis abundant protein Mrna, GenBank ID: DQ663481) was transferred into *Populus simonii* × *Populus nigra*. Among the eleven transgenic lines, one exhibited a dwarf phenotype compared to the wild type and other transgenic lines, named *dwf1*. To uncover the mechanisms underlying this phenotype, digital gene expression libraries were produced from *dwf1*, wild-type, and other normal transgenic lines, XL-5 and XL-6. Gene expression profile analysis indicated that *dwf1* had a unique gene expression pattern in comparison to the other two transgenic lines. Finally, a total of 1246 *dwf1*-unique differentially expressed genes were identified. These genes were further subjected to gene ontology and pathway analysis. Results indicated that photosynthesis and carbohydrate metabolism related genes were significantly affected. In addition, many transcription factors genes were also differentially expressed in *dwf1*. These various differentially expressed genes may be critical for dwarf mutant formation; thus, the findings presented here might provide insight for our understanding of the mechanisms of tree growth and development.

## 1. Introduction

Photosynthesis is a process used by plants and other organisms to convert light energy into chemical energy and store it in the bonds of sugars [1,2]. The process of photosynthesis takes place in the chloroplasts and employs the green pigment chlorophyll [3,4,5]. There are two parts to photosynthesis, namely, the light reaction and the dark reaction [6,7,8]. The light reaction occurs in the thylakoid membrane and converts light energy into chemical energy. This chemical reaction must, therefore, take place in the light. The light-harvesting complex is a complex of subunit proteins that make up part of a larger supercomplex, the photosystem, which is the functional unit of photosynthesis [9,10,11]. These complexes consist of proteins and photosynthetic pigments and surround a photosynthetic reaction center to focus energy, attained from photons absorbed by the pigment, toward the reaction center using Förster resonance energy transfer. Chlorophylls and carotenoids are important components of light-harvesting complexes in plants. The energy harvested via the light reaction is stored by forming ATP, a compound used by cells for energy storage.

The dark reaction, which takes place in the stroma within the chloroplast, converts CO_2_ to sugar. This reaction does not directly require light, but it does require the products of the light reaction (ATP and NADPH) [12,13]. The dark reaction involves the Calvin cycle, in which CO_2_ and energy from ATP are used to form sugar [14,15,16].

Monosaccharides are used as energy reserves in plants. Although a plethora of substrates such as proteins and lipids can be oxidized in plants, respiration tends to be dominated by carbohydrate oxidation through the glycolytic pathway and the tricarboxylic acid (TCA) or citric acid cycle. Carbohydrate is converted into pyruvate and malate through two major pathways, *i.e*., glycolysis and the oxidative pentose phosphate pathway. The primary roles of the pentose phosphate pathway are to generate NADPH for use in biosynthetic reactions, and to provide ribose 5-phosphate for nucleotide synthesis and erythrose 4-phosphate for the synthesis of shikimic acid derivatives [17].

In our previous study, the *Tamarix androssowii* *LEA* gene (lea IV; DQ663481; TaLEA) was introduced into *Populus simonii* × *Populus nigra* to improve salt tolerance [18,19]. During these studies, it was noted that one of these mutants displayed a dwarf phenotype compared with the other transgenic lines. Next generation (NG) sequencing methods have emerged as a cost-effective high-throughput approach to sequence a very large number of expressed genes, even in small experiments [20,21,22,23]. To uncover the mechanism of dwarf formation, digital gene expression (DGE) libraries were constructed and sequenced from the following lines: wild type, transgenic lines 5 and 6 (XL-5 and XL-6, *i.e.*, transgenic lines with normal phenotypes), and *dwf1*. In addition, we used gene ontology (GO) analysis to elucidate the functions of the genes that were found to be differentially expressed in the dwarf mutant.

## 2. Results

### 2.1. Isolation and Morphological Characterization of the dwf1 Phenotype

**Figure 1:**
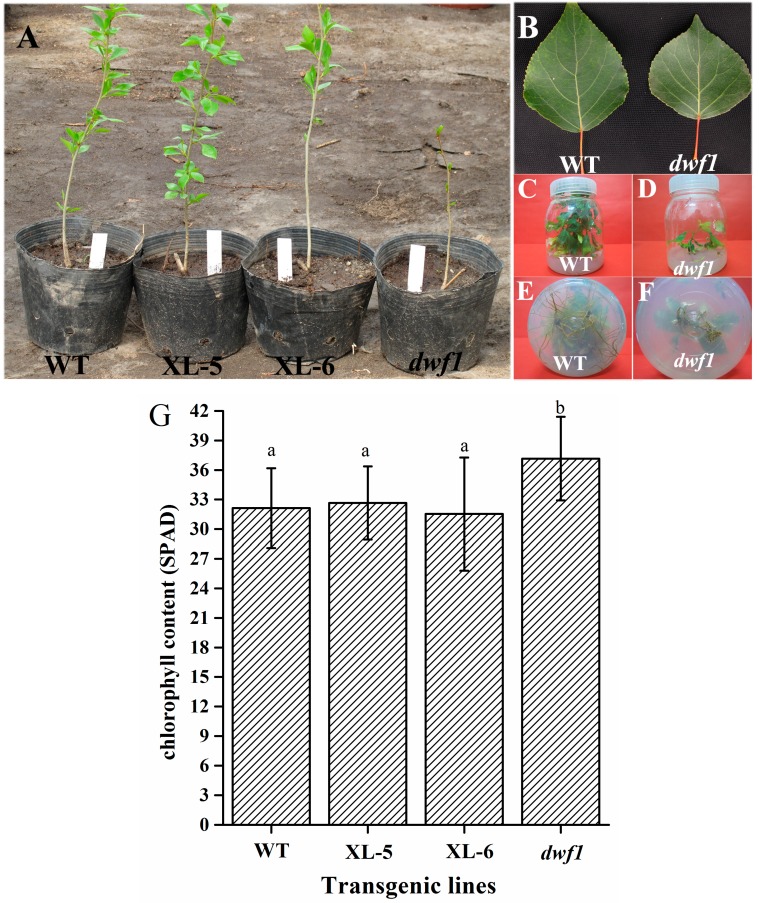
Phenotype of *dwf1*. (**A**) Seedlings of XL-5, XL-6, *dwf1* and wild-type. Under the same growth condition, *dwf1* exhibited a dwarf phenotype compared with the XL-5, XL-6 and wild-type; (**B**) Leaves of *dwf1* and wild-type. The leaf shape of *dwf1* was slightly affected; (**C**–**F**) Seedlings of *dwf1* and wild-type grown *in vitro*. (**F**) *Dwf1* exhibited reduced root biomass and fewer lateral roots compared with (**E**) wild-type; and (**G**) Chlorophyll content of XL-5, XL-6, *dwf1* and wild-type. Error bar indicates standard error (SD). Single factor ANOVA, followed by Tukey Kramer post-hoc test was employed in this study. Sixty leaves from six individuals of each transgenic line and wild-type were measured. Letters (a and b) on the graph denote significant differences (*p* < 0.05).

Among the 15 independent TaLEA transgenic lines, one grows extremely slowly compared to the wild type and the other transgenic lines, named *dwf1*, (Figure 1A–F). In addition to slow-growth, *dwf1* also exhibits dark green leaves. Chlorophyll content analysis indicated that *dwf1* had higher chlorophyll content (Figure 1G). These characteristics indicate that *dwf1* had multiple morphological defects (Figure 1).

### 2.2. Identification and Validation of Differentially Expressed Genes

**Figure 2:**
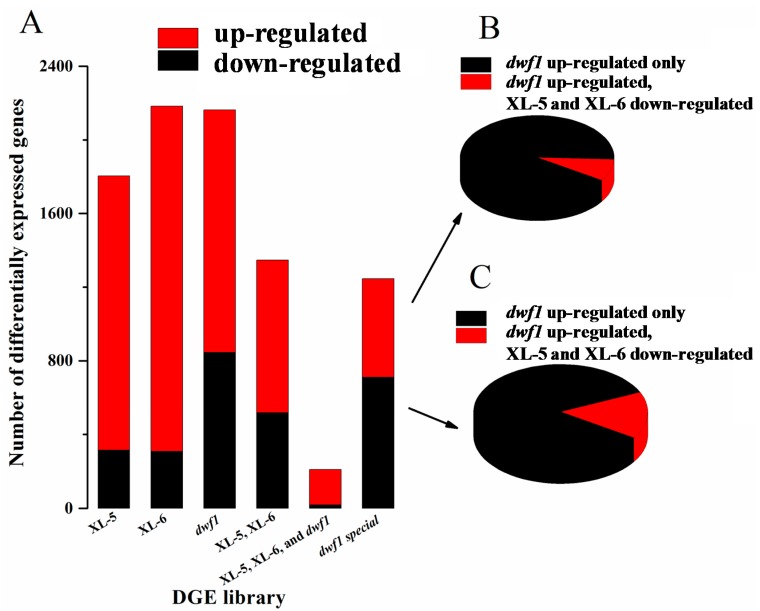
Distribution of differentially expressed genes. (**A**) Exact numbers of differently expressed genes. XL-5, XL-6, and *dwf1* indicate differently expressed genes in each sample in compared to wild-type (WT); “XL-5, XL-6” indicates common differently expressed genes in XL-5 and XL-6; “XL-5, XL-6, and *dwf1*” indicates common differently expressed genes in XL-5, XL-6 and *dwf1*; “*dwf1* special” indicates differentially expressed genes that were only identified in *dwf1*; (**B**) Expression patterns of *dwf1* special up-regulated genes in XL-5 and XL-6; (**C**) Expression patterns of *dwf1* special down-regulated genes in XL-5 and XL-6.

To identify genes showing significant changes in expression, we compared the levels of gene expression in XL-5, XL-6 and *dwf1* with the wild type. We identified 1804 (1489 upregulated and 315 downregulated), 2183 (1874 upregulated and 309 downregulated), and 2163 (1318 upregulated 315 downregulated), 2183 (1874 upregulated and 309 downregulated), and 2163 (1318 upregulated and 845 downregulated) differentially expressed genes in XL-5, XL-6 and *dwf1*, respectively. We further analyzed the intersection of differentially expressed genes. As shown in Figure 2A, most (947) of the differentially expressed genes (DEGs) in XL-5 and XL-6, shared similar expression patterns. In contrast, only 210 genes had similar expression patterns among these three transgenic lines. Instead, 1246 DEGs were *dwf1* unique. Of these *dwf1*-unique genes, 158 had opposite expression profiles between *dwf1* and the other two transgenic lines, XL-5 and XL-6. 1088 DEGs (491 upregulated, 597 downregulated) were only identified in *dwf1*. Detailed information is presented in Figure 2.

**Figure 3:**
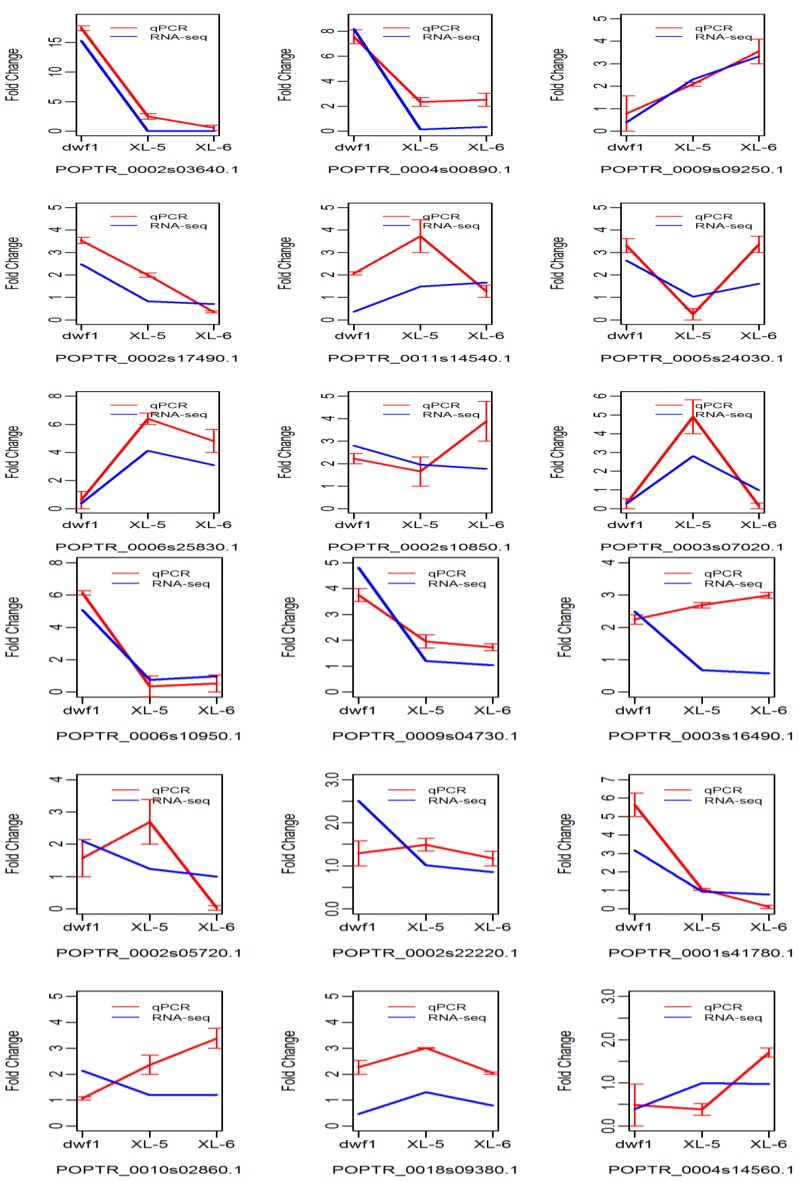
Validation of differentially expressed genes Fourteen genes showed similar expression patterns between digital gene expression data (blue lines) and RT-PCR (red lines) results.

To validation the digital gene expression (DGE), 18 differentially expressed genes were randomly selected for qPCR analysis. Fourteen of them showed similar expression patterns to those detected by DGE data (Figure 3), thus the results were reliable for identification of differentially expressed genes.

### 2.3. Gene Ontology Analysis

To reveal the molecular events underlying the transcriptome profile of *dwf1*, we further analyzed the 1246 *dwf1*-unique DEGs. GO category enrichment analysis was performed using an FDR (false discovery rate) adjusted *p*-value ≤0.05 as the cutoff (http://bioinfo.cau.edu.cn/agriGO/index.php) [24]. Table 1 lists the results of the GO enrichment analysis for the 1246 *dwf1*-specific differentially expressed genes. This analysis produced some significant findings. For biological processes, the most significantly enriched GO term was photosynthesis (GO: 0015979, *p*-value = 3.40 × 10^−^^10^). The GO terms included the following: photosynthesis, light harvesting (*p*-value = 6.20 × 10^−^^9^); photosynthesis, light reaction (*p*-value = 1.10 × 10^−^^8^); metabolic process (*p*-value = 1.60 × 10^−^^7^); cellular metabolic process (*p*-value = 2.20 × 10^−^^6^); generation of precursor metabolites and energy (*p*-value = 3.90 × 10^−^^6^); primary metabolic process (*p*-value = 2.10 × 10^−^^4^); cellular process (*p*-value = 2.80 × 10^−^^4^); and small molecule biosynthetic process (*p*-value = 4.90 × 10^−^^4^). The enriched GO terms in molecular function were catalytic activity (*p*-value = 7.00 × 10^−^^7^) and cofactor binding (*p*-value = 4.70 × 10^−^^5^). The enriched GO terms in cellular component were membrane (*p*-value = 1.20 × 10^−^^5^), photosystem (*p*-value = 0.00034), cell part (*p*-value = 0.00039), cell (*p*-value = 0.00039), photosynthetic membrane (*p*-value = 0.00064), thylakoid (*p*-value = 0.00089) and photosystem II (*p*-value = 0.002).

**Table 1:** GO enrichment results of the 1246 *dwf1*-specific differentially expressed genes.

GO Term	Ontology	Description	*p*-Value	FDR
GO:0015979	Biological process	photosynthesis	3.40 × 10^−^^10^	2.70 × 10^−7^
GO:0009765	Biological process	photosynthesis, light harvesting	6.20 × 10^−^^9^	2.50 × 10^−6^
GO:0019684	Biological process	photosynthesis, light reaction	1.10 × 10^−8^	3.00 × 10^−6^
GO:0008152	Biological process	metabolic process	1.60 × 10^−7^	3.20 × 10^−5^
GO:0044237	Biological process	cellular metabolic process	2.20 × 10^−6^	3.60 × 10^−4^
GO:0006091	Biological process	generation of precursor metabolites and energy	3.90 × 10^−6^	5.20 × 10^−4^
GO:0044238	Biological process	primary metabolic process	2.10 × 10^−4^	2.40 × 10^−2^
GO:0009987	Biological process	cellular process	2.80 × 10^−4^	2.80 × 10^−2^
GO:0044283	Biological process	small molecule biosynthetic process	4.90 × 10^−4^	4.30 × 10^−2^
GO:0003824	Molecular function	catalytic activity	7.00 × 10^−7^	4.30 × 10^−4^
GO:0048037	Molecular function	cofactor binding	4.70 × 10^−5^	0.014
GO:0016020	Cellular component	membrane	1.20 × 10^−5^	0.0015
GO:0009521	Cellular component	photosystem	0.00034	0.012
GO:0044464	Cellular component	cell part	0.00039	0.012
GO:0005623	Cellular component	cell	0.00039	0.012
GO:0034357	Cellular component	photosynthetic membrane	0.00064	0.016
GO:0009579	Cellular component	thylakoid	0.00089	0.019
GO:0009523	Cellular component	photosystem II	0.002	0.036

### 2.4. Pathway Analysis

Pathway analysis was performed using KEGG Automatic Annotation Server (KAAS) (http://www.genome.jp/tools/kaas/) [25]. The 1246 *dwf1*-specific differentially expressed genes were mapped to more than 100 pathways. As shown in Table 2, the results were coincident with the GO enrichment analysis. Numerous genes were mapped to photosynthesis, photosynthesis-antenna proteins, carbon fixation in photosynthetic organisms, and porphyrin and chlorophyll metabolism. Furthermore, carbohydrate metabolism was affected. The most representative pathways were glycolysis/gluconeogenesis, the citrate cycle (TCA cycle), the pentose phosphate pathway, starch and sucrose metabolism, and pyruvate metabolism (Table S1).

**Table 2:** Pathway analysis of the 1246 *dwf1*-specific differentially expressed genes.

Transcription ID	Description	Fold Change (*dwf1*/WT)	Fold Change (XL-5/WT)	Fold Change (XL-6/WT)
Photosynthesis				
POPTR_0004s03160.1	photosystem II oxygen-evolving enhancer protein 3	1.506534	−0.34166	0.307573
POPTR_0001s42970.1	photosystem II 10 kDa protein	1.19626	0.662679	0.540777
POPTR_0011s14540.1	photosystem II 10 kDa protein	−1.46887	0.568004	0.730908
POPTR_0005s24030.1	photosystem II PsbW protein	1.399979	0.036831	0.682095
POPTR_0002s05720.1	photosystem II Psb27 protein	1.072018	0.313421	−0.00522
POPTR_0002s24070.1	photosystem I subunit XI	2.55935	−0.33126	0.369094
POPTR_0007s04160.1	photosystem I subunit PsaN	1.029639	−0.93225	−0.78795
POPTR_0018s01830.1	F-type H^+^-transporting ATPase subunit b	1.012708	0.978216	0.799743
Photosynthesis—Antenna proteins				
POPTR_0014s17070.1	light-harvesting complex I chlorophyll *a*/*b* binding protein 3	1.312586	0.683911	0.509049
POPTR_0002s19010.1	light-harvesting complex II chlorophyll *a*/*b* binding protein 1	3.606024	−5.08746	0.640458
POPTR_0002s22220.1	light-harvesting complex II chlorophyll *a*/*b* binding protein 2	1.324373	0.019659	−0.22376
POPTR_0001s41780.1	light-harvesting complex II chlorophyll *a*/*b* binding protein 3	1.667425	−0.09416	−0.37883
POPTR_0016s12260.1	light-harvesting complex II chlorophyll *a*/*b* binding protein 4	1.374459	−0.40942	0.666267
POPTR_0006s10040.1	light-harvesting complex II chlorophyll *a*/*b* binding protein 4	1.20757	−0.35232	0.004115
POPTR_0001s21740.1	light-harvesting complex II chlorophyll *a*/*b* binding protein 6	1.023375	−0.57046	0.268189
Carbon fixation in photosynthetic organisms				
POPTR_0001s47210.1	fructose-bisphosphate aldolase, class I	1.599591	0.044778	0.18237
POPTR_0006s25830.1	aspartate aminotransferase, cytoplasmic	−1.42047	2.045731	1.631069
POPTR_0002s10850.1	phosphoenolpyruvate carboxykinase (ATP)	1.489119	0.967284	0.821559
POPTR_0012s01140.1	pyruvate kinase	−1.25054	0.074697	−0.16016
POPTR_0001s16300.1	alanine transaminase	−1.73081	1.165809	0.928917
POPTR_0003s07020.1	alanine transaminase	−1.91754	1.489279	−0.01162
POPTR_0010s02860.1	pyruvate, orthophosphate dikinase	1.094209	0.246454	0.270031
POPTR_0018s09380.1	malate dehydrogenase (oxaloacetate-decarboxylating) (NADP^+^)	−1.12428	0.386745	−0.34094
Porphyrin and chlorophyll metabolism				
POPTR_0004s14560.1	ferrochelatase	−1.35201	−0.00687	−0.03942
POPTR_0016s02570.1	magnesium-protoporphyrin IX monomethyl ester (oxidative) cyclase	1.057457	−0.85298	−0.25922
POPTR_0001s41370.1	protochlorophyllide reductase	1.416154	0.468741	0.329946
POPTR_0004s22680.1	pheophorbide a oxygenase	−1.54976	−0.0895	−0.36422
POPTR_0017s07890.1	glucuronosyltransferase	1.10191	0.043625	−0.20886
POPTR_0008s06270.2	glucuronosyltransferase	−2.35411	0.088461	0.088461
POPTR_0008s07270.2	ferritin heavy chain	−1.17819	0.509709	−0.74149
POPTR_0016s13270.3	ferritin heavy chain	−2.23599	2.993992	2.518902

### 2.5. Transcription Factors

Transcription factors are essential for the regulation of gene expression. They are vital for many important cellular processes. In this study, among the 1246 *dwf1*-unique DEGs, we identified 90 transcription factors, including 55 upregulated and 35 downregulated genes (Table 3). Among these 90 genes, 18 were MYB or MYB-related transcription factors, 12 were bHLH family proteins, eight were bZIPs, seven were NACs, five were C2H2 transcription factors, four were WRKYs, and the remaining genes included AP2, B3, RAV and others.

**Table 3:** Transcription factors in the 1246 *dwf1*-specific differentially expressed genes.

Transcription ID	Description	Fold Change (*dwf1*/WT)	Fold Change (XL-5/WT)	Fold Change (XL-6/WT)
POPTR_0002s03640.1	MYB_related	3.933054	−5.67243	−5.67243
POPTR_0002s26160.1	MYB_related	5.425953	−3.24793	−8.33539
POPTR_0004s00890.1	WRKY	3.033553	−2.90689	−1.53492
POPTR_0007s13000.1	MYB_related	1.605582	−2.71364	−1.86127
POPTR_0004s16320.1	MYB_related	2.125699	−2.05352	−3.3505
POPTR_0002s17460.1	MYB	1.117078	−1.29248	−1.32193
POPTR_0010s19400.1	bHLH	−1.56508	1.026258	1.111576
POPTR_0009s09250.1	C2H2	−1.35628	1.20679	1.732205
POPTR_0011s00390.1	MYB_related	−1.81444	1.290471	1.053793
POPTR_0014s09860.1	HD-ZIP	−4.14738	1.394444	1.900261
POPTR_0001s11380.1	HD-ZIP	−8.40939	1.803713	1.459432
POPTR_0001s41460.1	NAC	−4.2076	1.984222	1.11448
POPTR_0011s12400.1	NAC	−1.32851	1.996113	1.94709
POPTR_0018s00700.1	ERF	−2.9611	2.247418	2.369786
POPTR_0005s14120.1	G2-like	−1.03684	2.406678	1.683869
POPTR_0014s10190.4	bHLH	−6.08746	3.146157	3.697172
POPTR_0010s15280.1	bZIP	−5.67243	5.432828	5.434792
POPTR_0003s15060.1	ERF	4.18026	0.016874	0.31853
POPTR_0001s02650.2	LSD	3.752791	−0.97199	0.055495
POPTR_0009s11930.1	MYB_related	3.10598	0.016874	0.874469
POPTR_0005s25240.1	bHLH	2.614957	0.667425	0.244734
POPTR_0006s10950.1	WRKY	2.345377	−0.39896	−0.05247
POPTR_0009s04730.1	MYB	2.266787	0.265684	0.052089
POPTR_0001s27680.1	bHLH	2.185556	−0.56914	0.469682
POPTR_0003s19470.1	Trihelix	2.175913	0.556393	0.401473
POPTR_0009s04850.1	C2H2	2.04182	−0.3505	0.202044
POPTR_0014s10750.1	WRKY	1.96299	0.603699	0.342525
POPTR_0001s02450.1	bHLH	1.74573	0.094188	0.545172
POPTR_0005s22870.1	bHLH	1.739993	−0.20133	−0.43539
POPTR_0005s08510.1	NF-YB	1.712849	−0.9371	−0.46652
POPTR_0007s13020.1	bHLH	1.687038	−0.41627	−0.22301
POPTR_0007s12520.1	C2H2	1.684149	0.391269	0.468262
POPTR_0002s18290.1	NAC	1.56945	−0.63401	−0.81897
POPTR_0004s14510.1	MYB	1.558658	0.563823	−0.58928
POPTR_0015s05430.1	MYB_related	1.543734	0.579224	0.903746
POPTR_0003s05010.1	bHLH	1.502954	0.365995	0.91663
POPTR_0008s23260.1	ERF	1.487078	0.257027	−0.46781
POPTR_0014s10700.1	bHLH	1.390702	0.163825	0.219781
POPTR_0001s13380.1	NAC	1.340465	0.601475	0.417288
POPTR_0019s04820.1	G2-like	1.337559	−0.92946	−0.24244
POPTR_0008s13980.1	C2H2	1.322666	0.078973	0.660438
POPTR_0020s00320.1	CO-like	1.31853	0.694946	0.934017
POPTR_0003s16490.1	NAC	1.315454	−0.56824	−0.79463
POPTR_0002s17490.1	Dof	1.307035	−0.27635	−0.50763
POPTR_0003s03670.1	NAC	1.295921	0.583914	0.757562
POPTR_0018s12990.1	YABBY	1.295671	0.696682	0.971257
POPTR_0019s14460.1	WRKY	1.270407	0.876839	0.972344
POPTR_0002s00980.1	WOX	1.257934	0.452272	0.193424
POPTR_0013s03800.1	bZIP	1.256512	−0.6204	0.741133
POPTR_0017s02990.1	MYB	1.253047	0.263558	0.822986
POPTR_0013s05670.1	G2-like	1.206943	−0.29725	0.336728
POPTR_0007s07480.1	TCP	1.186807	0.725161	0.680275
POPTR_0001s44230.1	bHLH	1.170218	0.713709	0.237438
POPTR_0003s20320.1	bZIP	1.161617	0.485959	0.893658
POPTR_0008s11600.1	RAV	1.143492	0.865699	0.846949
POPTR_0016s00780.1	NF-YB	1.13943	0.700983	0.789505
POPTR_0013s15280.1	bZIP	1.132915	0.541894	0.886952
POPTR_0004s16660.1	TALE	1.1283	0.562619	0.236414
POPTR_0002s25960.4	G2-like	1.117078	0.431306	0.101913
POPTR_0007s01230.1	G2-like	1.110973	−0.85528	0.640587
POPTR_0010s19520.1	C3H	1.108934	0.950292	−0.08473
POPTR_0003s13120.1	TALE	1.088253	−0.3639	−0.2824
POPTR_0016s11980.1	MYB	1.037428	0.245169	−0.25188
POPTR_0003s12990.1	MYB_related	1.014075	0.018072	0.946484
POPTR_0016s08470.2	MYB_related	1.01281	0.124458	0.919683
POPTR_0012s07270.1	HD-ZIP	1.000161	0.522719	0.909557
POPTR_0018s13860.1	bHLH	−1.02003	0.086354	−0.70214
POPTR_0009s06540.2	NF-YA	−1.03937	0.522171	0.527314
POPTR_0013s11300.1	C2H2	−1.05068	-0.54184	−0.86987
POPTR_0014s08520.1	MYB_related	−1.10493	−0.16666	0.093567
POPTR_0002s17680.1	HD-ZIP	−1.18914	0.815691	0.971304
POPTR_0007s10490.1	MYB	−1.20875	0.415756	0.183596
POPTR_0014s02260.1	MYB	−1.22876	−0.67693	−0.58973
POPTR_0002s09480.1	ERF	−1.26701	0.071166	−0.84495
POPTR_0004s14900.1	B3	−1.35861	−0.27417	−0.5989
POPTR_0002s12710.1	bZIP	−1.39873	0.697847	0.76797
POPTR_0001s08990.1	HSF	−1.41814	0.198258	0.129962
POPTR_0008s04490.1	AP2	−1.42131	−0.31532	−0.2299
POPTR_0005s05330.1	Trihelix	−1.43381	0.303158	−0.03779
POPTR_0008s11120.2	bHLH	−1.4783	−0.16036	−0.73637
POPTR_0001s38120.1	HD-ZIP	−1.5621	−0.50735	0.562355
POPTR_0002s16870.1	bZIP	−1.65421	0.202417	−0.16211
POPTR_0005s19580.1	GATA	−1.70355	0.247976	−0.30077
POPTR_0009s10400.1	bZIP	−1.81729	−0.26219	−0.56068
POPTR_0014s08990.1	bZIP	−1.83325	0.040062	−0.14179
POPTR_0002s15360.1	TCP	−2.15325	−0.22441	−0.04544
POPTR_0011s11620.1	ERF	−2.35326	0.870376	−0.53229
POPTR_0014s00780.1	GRF	−2.83592	0.910389	0.983744
POPTR_0002s08150.1	NAC	−3.0591	−0.10704	−0.05318
POPTR_0004s03310.1	MYB	−4.32769	0.526308	−0.6206

## 3. Discussion

### 3.1. Dwf1 Showed a Unique Gene Expression Profile Compared to the Other Transgenic Lines

In our previous study, a *TaLEA* gene (GenBank: DQ663481.1) was introduced into poplar (*Populus simonii* × *P. nigra*) by *Agrobacterium* tumefaciens-mediated transformation [18,19]. All of the 11 transgenic lines grew normally except *dwf1*, so we analyzed the transcriptional differences among XL-5, XL-6, *dwf1* and wild type. As expected, XL-5 and XL-6 shared more DEGs (Figure 2), and XL-5, XL-6 and *dwf1* shared fewer. Instead, a larger number of *dwf1*-unique DEGs were identified (1246 genes). More than 150 genes showed a contrasting expression profile in *dwf1* compared to XL-5 and XL-6. The unique expression pattern of *dwf1* indicated that the molecular events occurring in *dwf1* were different from the other transgenic lines. Indeed, further analysis of the *dwf1*-unique DEGs may provide useful insights for further investigation of the molecular mechanisms underlying this mutant.

### 3.2. T-DNA-Affected Genes Might Play a Role in Dwarfism Formation

As only *dwf1* exhibited a dwarf phenotype, the T-DNA insertion sites of *dwf1* may play a role in the formation of this mutant. The T-DNA-affected genes or the interaction between *TaLEA* and T-DNA-affected genes may have affected the *dwf1* gene expression pattern. In our previous work, two T-DNA insertion sites and a T-DNA-affected gene (*RAV*) were isolated, and qRT-PCR analysis showed that *RAV* was specifically upregulated in *dwf1* [18,19]. Genes associated with starch, sucrose, galactose and glycerolipid metabolism were different expressed in *dwf1* compared to wild-type. These results were consistent with this study, however only *dwf1* and wild-type were analyzed in previous studies and it was impossible to identity *dwf1*-unique DEGs. In order to overcome this difficulty, we sequenced other transgenic lines (XL-5 and XL-6), allowing more useful *dwf1*-unique DEGs relevant for uncovering the mechanism of dwarfism.

### 3.3. Genes Involved in Photosynthesis and Photosynthesis-Antenna Proteins

Many genes involved in photosynthesis and photosynthesis-antenna proteins were specifically differentially expressed in *dwf1* (Table 2). Photosystem II PsbW protein (POPTR_0005s24030.1), photosystem II Psb27 protein (POPTR_0002s05720.1), photosystem II 10 kDa protein (POPTR_0001s42970.1) and photosystem II 10 kDa protein (POPTR_0011s14540.1) were differentially expressed in *dwf1*. PsbW, Psb27, and photosystem II 10 kDa protein are the main components of photosystem II, and photosystem II is the first protein complex in the light-dependent reaction. The process of photosynthesis requires a variety of energy transducing protein complexes that transport electrons and pump protons, leading to the formation of ATP and NADPH. Photosystem II (PS II) is one of these protein complexes, and it has been the focus of many studies. PS II transfers electrons from water to plastoquinone, and this process generates a pH gradient. Plastoquinone (PQ) carries electrons from PS II to the cytochrome bf complex. This complex traps light and uses it to reduce the QB plastoquinone and to synthesize molecular oxygen from water [26,27,28,29]. Photosystem I (PS I) is the second photosystem in plants, and it contains many subunits. PS I subunit XI (POPTR_0002s24070.1) and PS I subunit PsaN (POPTR_0007s04160.1) were found to be specifically upregulated in *dwf1* (Table 2). ATPases are a class of enzymes that catalyze the decomposition of ATP into ADP and a free phosphate ion. This dephosphorylation reaction releases energy [30,31]. In this study, an F-type H^+^-transporting ATPase subunit b (POPTR_0018s01830.1) was found to be specifically upregulated in *dwf1*.

The antennae of plants consist of a large number of protein-bound chlorophyll molecules that absorb photons and transfer their energy to the reaction center. The antennae of plants consist of an inner part and an outer part. The outer part, formed by the light harvesting complexes (LHCs), collects light. The inner part of the antenna, consisting of core complexes, is an integral constituent of the reaction centers. LHCs are formed by polypeptides, which bind chl-*a*, chl-*b*, xanthophylls and carotenes [32,33]. In this study, seven LHC chlorophyll *a*/*b* binding proteins were found to be specifically upregulated in *dwf1*, including six LHC II chlorophyll *a*/*b* binding proteins and one LHC I chlorophyll *a*/*b* binding protein (Table 2).

**Figure 4:**
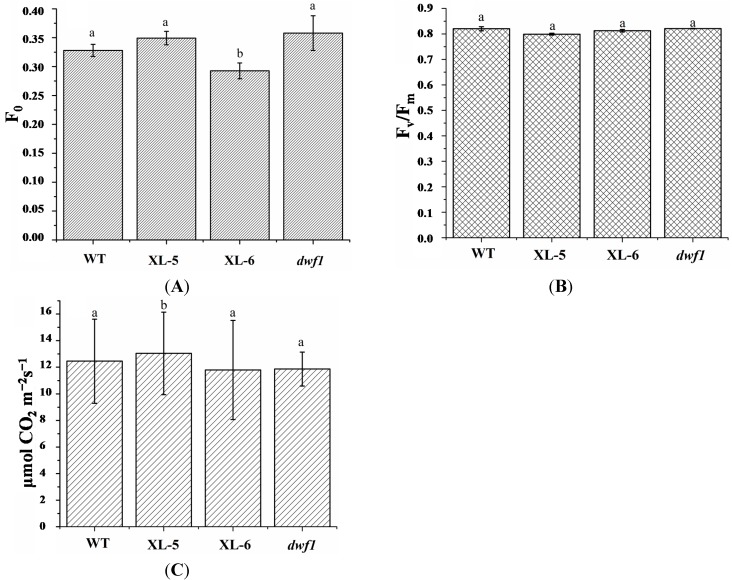
Photosynthetic fluorescence parameters chlorophyll fluorescence parameters (F_0_ and F_v_/F_m_) and photosynthetic efficiency of XL-5, XL-6, *dwf1*, and WT. Results are presented as the mean ± SD of three independent experiments. (**A**) F_0_; (**B**) F_v_/F_m_; (**C**) photosynthetic efficiency. Single factor Anova, followed by Tukey Kramer post-hoc test was employed in this study. Thirty leaves from six individuals of each transgenic line and wild-type were measured. Letters on the graph denote significant differences (*p* < 0.05).

The altered expression profiles, especially the upregulated expression profile of these photosynthesis-related genes, indicate that *dwf1* might have an increased photosynthetic efficiency. We therefore measured the photosynthetic rates and photosynthetic fluorescence parameters of wild type, XL-5, XL-6 and *dwf1*. Unexpectedly, the altered gene expression in *dwf1* did not result in any detectable differences in photosynthetic efficiency or photosynthetic fluorescence parameters (Figure 4).

### 3.4. Genes Involved in Carbohydrate Metabolism

According to the DGE data, the expression of numerous genes involved in carbohydrate metabolism was specifically altered in *dwf1*, including many key enzymes involved in glycolysis/gluconeogenesis, the citrate cycle (TCA cycle), pentose phosphate pathway, pentose and glucuronate interconversions, fructose and mannose metabolism, galactose metabolism, starch and sucrose metabolism, amino sugar and nucleotide sugar metabolism, and pyruvate metabolism (Table S1). Glucose-6-phosphate isomerase is an enzyme that catalyzes the conversion of glucose-6-phosphate into fructose 6-phosphate in the second step of glycolysis. Glucose-6-phosphate isomerase is also involved in the pentose phosphate pathway and starch and sucrose metabolism [34,35,36]. In this study, a gene that encodes a glucose-6-phosphate isomerase was found to be specifically downregulated in *dwf1*. Phosphoglucomutase is an enzyme that transfers a phosphate group on the α-d-glucose monomer from the 1' to the 6' position in the forward direction or the 6' to the 1' position in the reverse direction. This reaction is involved in glycolysis/gluconeogenesis, the pentose phosphate pathway, galactose metabolism, and starch and sucrose metabolism [37,38,39]. In this study, two genes that encode phosphoglucomutase were found to be specifically downregulated in *dwf1*. Glucose is a major product of photosynthesis, and is stored mainly in the form of starch granules in plastids such as chloroplasts, and especially in amyloplasts. Starch biosynthesis in higher plants is catalyzed by four classes of enzymes, including ADP-Glc pyrophosphorylase (AGPase), starch synthase (SS), starch branching enzyme (BE), and starch debranching enzyme. SS elongates glucans by adding Glc residues from ADP-Glc to the glucan nonreducing ends through α-1,4 linkages. In this study, a starch synthase was found to be specifically upregulated in *dwf1*.

### 3.5. Transcription Factors

Transcription factors are shaped so precisely that they are able to bind preferentially to the regulatory regions of only a few genes out of the thousands present in a cell’s DNA. Transcription factors are involved in almost all biological processes, such as development, responses to intercellular signals, responses to the environment, and pathogenesis. In this study, 90 transcription factors were found to be specifically differentially expressed in *dwf1*. Among these genes, 17 displayed an expression profile in *dwf1* that was the opposite of that observed in XL-5 and XL-6, and 73 were found to be differentially expressed in *dwf1*, while the expression of these genes was not altered in XL-5 or XL-6 (Table 3).

MYB transcription factors are known to play an important role in the control of phenylpropanoid metabolism [40,41]. Plant MYB proteins also play a role in hormonal responses during seed development and germination. In this study, more than ten MYB and MYB-related transcription factors were found to be differentially expressed. We also found that WRKY, NAC, bZIP, AP2, RAV and other transcription factors were differentially expressed in *dwf1* [42,43,44,45]. All of these differentially expressed genes may play a vital role in dwarf mutant formation.

## 4. Experimental Section

### 4.1. Plant Growth Conditions and Transgenic Plants

The transgenic poplar (*Populus simonii* × *P. nigra*) lines were produced as previously described [46]. The seedlings were planted in a mixture of turfy peat and sand (2:1 *v*/*v*) and transferred to a greenhouse at 75% relative humidity and a constant temperature of 24 °C. All wild-type and transgenic plants used for analysis were three months old. The third and fourth young leaves, counted from top, were harvested from six plants of each transgenic line and wild-type. Leaves harvested from two plants of each transgenic line and wild-type were pooled together as one replicate. The leaves were immediately immersed in liquid nitrogen and stored at −70 °C for subsequent RNA extraction.

### 4.2. Chlorophyll Content

A commercial chlorophyll meter (SPAD-502, Spectrum Technologies, Chicago, IL, USA) was used to estimate the chlorophyll contents of leaves. Six independent plants from each transgenic line and the wild type were randomly selected. And then ten leaves of each plant were measured.

### 4.3. RNA Isolation and RNA-seq Library Preparation

Total RNA was isolated from each sample using the CTAB method [47]. All RNA samples were quantified and examined spectrophotometrically for protein contamination (A260/A280 ratios) and reagent contamination (A260/A230 ratios). Extracted RNA samples were selected based on the 28S/18S rRNA band intensity (2:1), spectroscopic A260/A280 readings between 1.8 and 2.0, and A260/A230 readings greater than 1.5. According to the protocol, we generate three libraries for each sample. Subsequently, sequencing by synthesis (SBS) was performed using four types of nucleotides identified by labeling with four different colors.

### 4.4. Data Analysis

Raw reads were filtered through the Illumina pipeline. After that, clean tags were mapped to poplar reference sequences (http://www.phytozome.net/poplar) [48] using SOAP (Short Oligonucleotide Analysis Package) [49] A library was generated containing all possible fragments CATG + 17 bases in length among the reference gene sequences. All clean tags were mapped to the reference sequences, allowing for only 1 bp mismatch. Clean tags that mapped to multiple genes were discarded. The number of unambiguous clean tags was calculated for each gene and normalized to the number of transcripts per million clean tags (TPM) [50,51].

Differential expressed genes (DEGs) were obtained by pairwise comparison of transgenic and WT libraries using edgeR/Bioconductor [24]. The significance of DEGs is based on a false discovery rate (FDR) of 0.05 and a log2 fold change of 1.

GO analysis was performed for functional categorization of differentially expressed genes using agriGO software (http://bioinfo.cau.edu.cn/agriGO/index.php) [52], and the *p*-values were corrected by applying the FDR correction to control falsely rejected hypotheses during GO analysis. The hypergeometric test was used as the statistical test, and an FDR corrected *p-*value ≤0.05 was used as the cutoff value [25].

Pathway analysis was performed using KEGG Automatic Annotation Server (KAAS) (http://www.genome.jp/tools/kaas/) [25]. Differentially expressed genes were mapped to terms in the Kyoto Encyclopedia of Genes and Genomes (KEGG) database.

### 4.5. Real-Time RT-PCR

cDNA was fist synthesized from purified RNA using Reverse Transcription Kit (Qiagen, Hilden, Germany), and then 1:10 diluted using Rnase-free water. Quantitative PCR was performed using 1 μL of diluted cDNA as a template in a 20 μL of SYBR Green (Toyobo, Osaka, Japan) mixed reaction. The relative expression levels of the selected genes were calculated using the relative 2^−ΔΔ*C*t^ method [53]. α*-tubulin* and *actin* served as the internal reference genes. Results represent mean standard deviation of the three experimental replicates.

## 5. Conclusions

This study has demonstrated the usefulness of the digital gene expression (DGE) approach to identify differentially expression genes between *dwf1*, XL-5, XL-6, and WT. The dwarf mutant was found to specifically exhibit differential expression of numerous genes involved in photosynthesis and photosynthesis-antenna proteins, genes involved in carbohydrate metabolism, and genes encoding transcription factors. These findings might provide a strong basis for future research on dwarf mutant formation.

## Supplementary Materials

Supplementary Tables can be found at http://www.mdpi.com/1422-0067/15/9/15603/s1.

## Supplementary Files

Supplementary File 1
