# Supplementary material for: Comparative Genomic Analysis of Transgenic Poplar Dwarf Mutant Reveals Numerous Differentially Expressed Genes Involved in Energy Flow
Source: Int J Mol Sci. 2014 Sep 3;15(9):15603–21. doi: 10.3390/ijms150915603 (PMC4200758; doi:10.3390/ijms150915603)
Supplement: Supplementary File 1 [file ijms-15-15603-s001.pdf]

Supplementary Information

Table S1. Pathway analysis of the 1246 *dwf1*-specific differentially expressed genes.

Gene ID	Description	Fold Change (*dwf1*/WT)	Fold Change (XL-5/WT)	Fold Change (XL-6/WT)
**Carbohydrate Metabolism**				
**Glycolysis/Gluconeogenesis**				
POPTR_0008s11770.1	Glucose-6-phosphate isomerase	−1.7382	0.512146	0.168727
POPTR_0001s47210.1	Fructose-bisphosphate aldolase, class I	1.599591	0.044778	0.18237
POPTR_0011s05190.3	Probable phosphoglycerate mutase	−2.11069	−0.34562	−0.9738
POPTR_0012s01140.1	Pyruvate kinase	−1.25054	0.074697	−0.16016
POPTR_0016s12760.1	Pyruvate decarboxylase	2.664081	0.021062	0.371969
POPTR_0012s08010.1	Aldehyde dehydrogenase (NAD^+)	−1.41556	0.479957	−0.21366
POPTR_0014s13710.1	Acetyl-CoA synthetase	−1.337	0.154552	−0.26532
POPTR_0017s11660.1	Aldose 1-epimerase	2.770518	0.016874	0.73016
POPTR_0010s11970.1	Phosphoglucomutase	−1.25266	−0.35581	0.074064
POPTR_0012s14030.1	Phosphoglucomutase	−1.15872	−0.68468	−0.93596
POPTR_0002s10850.1	Phosphoenolpyruvate carboxykinase (ATP)	1.489119	0.967284	0.821559
**Citrate cycle (TCA cycle)**				
POPTR_0014s15280.1	2-Oxoglutarate dehydrogenase E2 component (dihydrolipoamide succinyltransferase)	−1.63733	0.076435	0.170827
POPTR_0002s26120.1	Succinyl-CoA synthetase β subunit	−1.29244	−0.38517	−0.3497
POPTR_0007s12750.1	Succinate dehydrogenase (ubiquinone) flavoprotein subunit	−1.83751	0.519356	0.309149
POPTR_0002s10850.1	Phosphoenolpyruvate carboxykinase (ATP)	1.489119	0.967284	0.821559
**Pentose phosphate pathway**				
POPTR_0008s11770.1	Glucose-6-phosphate isomerase	−1.7382	0.512146	0.168727
POPTR_0013s00660.1	Glucose-6-phosphate 1-dehydrogenase	−1.26949	−0.18314	0.374822
POPTR_0015s00960.1	6-Phosphogluconolactonase	2.022223	0.168877	0.971431
POPTR_0010s11970.1	Phosphoglucomutase	−1.25266	−0.35581	0.074064
POPTR_0012s14030.1	Phosphoglucomutase	−1.15872	−0.68468	−0.93596
POPTR_0001s47210.1	Fructose-bisphosphate aldolase, class I	1.599591	0.044778	0.18237

Table S1. *Cont.*

Gene ID	Description	Fold Change (*dwf1*/WT)	Fold Change (XL-5/WT)	Fold Change (XL-6/WT)
**Pentose and glucuronate interconversions**				
POPTR_0010s23570.1	Pectate lyase	2.901222	0.436099	0.055495
POPTR_0017s07890.1	Glucuronosyltransferase	1.10191	0.043625	−0.20886
POPTR_0008s06270.2	Glucuronosyltransferase	−2.35411	0.088461	0.088461
POPTR_0012s08010.1	Aldehyde dehydrogenase (NAD^+)	−1.41556	0.479957	−0.21366
**Fructose and mannose metabolism**				
POPTR_0007s01850.1	Fructokinase	1.462343	0.1181	0.649348
POPTR_0008s06050.1	Mannose-1-phosphate guanylyltransferase	1.558274	0.760752	0.460438
POPTR_0001s47210.1	Fructose-bisphosphate aldolase, class I	1.599591	0.044778	0.18237
**Galactose metabolism**				
POPTR_0010s11970.1	Phosphoglucomutase	−1.25266	−0.35581	0.074064
POPTR_0012s14030.1	Phosphoglucomutase	−1.15872	−0.68468	−0.93596
POPTR_0018s12670.1	Raffinose synthase	−3.291	2.639741	1.162461
POPTR_0006s22710.1	β-Fructofuranosidase	1.276758	0.648028	0.961749
**Starch and sucrose metabolism**				
POPTR_0006s22710.1	β-Fructofuranosidase	1.276758	0.648028	0.961749
POPTR_0015s04280.2	β-Glucosidase	2.422972	−0.97199	0.281771
POPTR_0011s06910.3	α, α-trehalose-phosphate synthase (UDP-forming)	−1.0224	0.047291	0.655618
POPTR_0003s14390.1	α, α-trehalase	−1.365	2.338802	1.901222
POPTR_0017s07890.1	Glucuronosyltransferase	1.10191	0.043625	−0.20886
POPTR_0008s06270.2	Glucuronosyltransferase	−2.35411	0.088461	0.088461
POPTR_0001s32730.1	UDP-glucuronate 4-epimerase	1.53248	−0.26328	0.608462
POPTR_0010s11970.1	Phosphoglucomutase	−1.25266	−0.35581	0.074064
POPTR_0012s14030.1	Phosphoglucomutase	−1.15872	−0.68468	−0.93596
POPTR_0008s11770.1	Glucose-6-phosphate isomerase	−1.7382	0.512146	0.168727
POPTR_0007s01850.1	Fructokinase	1.462343	0.1181	0.649348
POPTR_0017s12040.1	Starch synthase	1.133033	0.256905	0.651671

Table S1. *Cont.*

Gene ID	Description	Fold Change (*dwf1*/WT)	Fold Change (XL-5/WT)	Fold Change (XL-6/WT)
**Starch and sucrose metabolism**				
POPTR_0006s11620.1	1,4-α-Glucan branching enzyme	−1.70221	0.687539	−0.11443
POPTR_0005s27240.1	1,4-α-Glucan branching enzyme	−2.06114	1.257613	1.590735
**Amino sugar and nucleotide sugar metabolism**				
POPTR_0009s14420.1	Chitinase	2.318006	0.527247	0.907326
POPTR_0008s07890.1	Hexosaminidase	1.425299	−0.2544	0.698225
POPTR_0003s07230.1	UDP-*N*-acetylglucosamine pyrophosphorylase	−1.56059	0.507552	0.630126
POPTR_0007s01850.1	Fructokinase	1.462343	0.1181	0.649348
POPTR_0011s15960.1	UDP-arabinose 4-epimerase	1.676303	0.238467	0.884719
POPTR_0008s11770.1	Glucose-6-phosphate isomerase	−1.7382	0.512146	0.168727
POPTR_0010s11970.1	Phosphoglucomutase	−1.25266	−0.35581	0.074064
POPTR_0012s14030.1	Phosphoglucomutase	−1.15872	−0.68468	−0.93596
POPTR_0001s32730.1	UDP-glucuronate 4-epimerase	1.53248	−0.26328	0.608462
POPTR_0008s06050.1	Mannose-1-phosphate guanylyltransferase	1.558274	0.760752	0.460438
**Pyruvate metabolism**				
POPTR_0014s13710.1	Acetyl-CoA synthetase	−1.337	0.154552	−0.26532
POPTR_0012s01140.1	Pyruvate kinase	−1.25054	0.074697	−0.16016
POPTR_0012s08010.1	Aldehyde dehydrogenase (NAD^+)	−1.41556	0.479957	−0.21366
POPTR_0016s06480.1	D-lactate dehydrogenase (cytochrome)	−1.33053	0.130789	−0.33533
POPTR_0018s09380.1	Malate dehydrogenase (oxaloacetate-decarboxylating)($NADP^+$)	−1.12428	0.386745	−0.34094
POPTR_0002s10850.1	Phosphoenolpyruvate carboxykinase (ATP)	1.489119	0.967284	0.821559
POPTR_0010s02860.1	Pyruvate,orthophosphate dikinase	1.094209	0.246454	0.270031
POPTR_0005s08480.1	2-Isopropylmalate synthase	−1.57022	−0.57116	−0.58386
**Energy Metabolism**				
**Oxidative phosphorylation**				
POPTR_0002s11860.1	NADH dehydrogenase (ubiquinone) Fe-S protein 4	−4.06117	0.553126	−0.04615
POPTR_0010s06750.1	NADH dehydrogenase (ubiquinone) flavoprotein 1	−1.41318	0.23172	0.185594

Table S1. *Cont.*

Gene ID	Description	Fold Change (*dwf1*/WT)	Fold Change (XL-5/WT)	Fold Change (XL-6/WT)
**Energy Metabolism**				
**Oxidative phosphorylation**				
POPTR_0013s08220.1	NADH dehydrogenase (ubiquinone) 1 α/β subcomplex 1	−1.42463	−0.23806	−0.25433
POPTR_0009s12750.1	NADH dehydrogenase (ubiquinone) 1 β subcomplex 9	−2.0525	−0.04824	−0.37689
POPTR_0007s12750.1	Succinate dehydrogenase (ubiquinone) flavoprotein subunit	−1.83751	0.519356	0.309149
**Energy Metabolism**				
**Oxidative phosphorylation**				
POPTR_0018s01830.1	F-type H^+-transporting ATPase subunit b	1.012708	0.978216	0.799743
POPTR_0001s33930.1	V-type H^+-transporting ATPase subunit D	−1.27109	−0.31451	−0.14923
POPTR_0006s28990.1	H^+-transporting ATPase	1.714807	−0.17158	0.774057
POPTR_0015s07710.1	H^+-transporting ATPase	−1.2267	0.153242	−0.17539
POPTR_0013s01080.1	Inorganic pyrophosphatase	1.468353	0.502301	0.732205
POPTR_0006s08250.1	Inorganic pyrophosphatase	−1.05129	−0.16531	−0.24767
**Photosynthesis**				
POPTR_0004s03160.1	Photosystem II oxygen-evolving enhancer protein 3	1.506534	−0.34166	0.307573
POPTR_0001s42970.1	Photosystem II 10 kDa protein	1.19626	0.662679	0.540777
POPTR_0011s14540.1	Photosystem II 10 kDa protein	−1.46887	0.568004	0.730908
POPTR_0005s24030.1	Photosystem II PsbW protein	1.399979	0.036831	0.682095
POPTR_0002s05720.1	Photosystem II Psb27 protein	1.072018	0.313421	−0.00522
POPTR_0002s24070.1	Photosystem I subunit XI	2.55935	−0.33126	0.369094
POPTR_0007s04160.1	Photosystem I subunit PsaN	1.029639	−0.93225	−0.78795
POPTR_0018s01830.1	F-type H^+-transporting ATPase subunit b	1.012708	0.978216	0.799743
**Photosynthesis-antenna proteins**				
POPTR_0014s17070.1	Light-harvesting complex I chlorophyll a/b binding protein 3	1.312586	0.683911	0.509049
POPTR_0002s22220.1	Light-harvesting complex II chlorophyll a/b binding protein 2	1.324373	0.019659	−0.22376
POPTR_0001s41780.1	Light-harvesting complex II chlorophyll a/b binding protein 3	1.667425	−0.09416	−0.37883
POPTR_0006s10040.1	Light-harvesting complex II chlorophyll a/b binding protein 4	1.20757	−0.35232	0.004115

Table S1. *Cont.*

Gene ID	Description	Fold Change (*dwf1*/WT)	Fold Change (XL-5/WT)	Fold Change (XL-6/WT)
**Photosynthesis-antenna proteins**				
POPTR_0016s12260.1	Light-harvesting complex II chlorophyll a/b binding protein 4	1.374459	−0.40942	0.666267
POPTR_0001s21740.1	Light-harvesting complex II chlorophyll a/b binding protein 6	1.023375	−0.57046	0.268189
**Photosynthesis proteins**				
**Carbon fixation in photosynthetic organisms**				
POPTR_0001s47210.1	Fructose-bisphosphate aldolase, class I	1.599591	0.044778	0.18237
POPTR_0006s25830.1	Aspartate aminotransferase, cytoplasmic	−1.42047	2.045731	1.631069
POPTR_0002s10850.1	Phosphoenolpyruvate carboxykinase (ATP)	1.489119	0.967284	0.821559
POPTR_0012s01140.1	Pyruvate kinase	−1.25054	0.074697	−0.16016
POPTR_0010s02860.1	Pyruvate,orthophosphate dikinase	1.094209	0.246454	0.270031
POPTR_0018s09380.1	Malate dehydrogenase (oxaloacetate-decarboxylating)($NADP^+$)	−1.12428	0.386745	−0.34094
**Lipid Metabolism**				
**Fatty acid metabolism**				
POPTR_0018s08940.1	Enoyl-CoA hydratase/3-hydroxyacyl-CoA dehydrogenase	−2.03577	0.31825	0.295404
POPTR_0006s10270.1	Acyl-CoA oxidase	−1.0383	−0.09723	−0.69011
POPTR_0001s32900.3	Long-chain acyl-CoA synthetase	−2.51488	−0.56873	−0.64835
POPTR_0002s08450.1	Long-chain acyl-CoA synthetase	−1.28507	−0.83726	−0.80057
POPTR_0010s10050.3	Long-chain acyl-CoA synthetase	−1.35147	0.105243	0.13351
POPTR_0012s08010.1	Aldehyde dehydrogenase (NAD^+)	−1.41556	0.479957	−0.21366
**Nucleotide Metabolism**				
**Purine metabolism**				
POPTR_0010s11970.1	Phosphoglucomutase	−1.25266	−0.35581	0.074064
POPTR_0012s14030.1	Phosphoglucomutase	−1.15872	−0.68468	−0.93596
POPTR_0004s23390.1	Phosphoribosylamine-glycine ligase	−1.09954	−0.09954	−0.68706
POPTR_0010s20790.1	Phosphoribosylformylglycinamidine synthase	−1.3279	−0.25124	−0.23671
POPTR_0008s08230.1	IMP dehydrogenase	#NUM!	−0.98126	0.130629
POPTR_0012s01140.1	Pyruvate kinase	−1.25054	0.074697	−0.16016

Table S1. *Cont.*

Gene ID	Description	Fold Change (*dwf1*/WT)	Fold Change (XL-5/WT)	Fold Change (XL-6/WT)
**Nucleotide Metabolism**				
**Purine metabolism**				
POPTR_0004s04320.1	DNA-directed RNA polymerase II subunit RPB2	−1.16741	1.003588	1.398599
POPTR_0015s15080.1	Heat-stable enterotoxin receptor	2.2849	−2.70044	#NUM!
POPTR_0010s22250.1	Adenylate kinase	1.850352	0.397964	0.978626
POPTR_0018s12140.1	Adenylate kinase	−1.21892	0.415146	−0.65949
POPTR_0003s08190.2	Ureidoglycine aminohydrolase	−1.18759	0.625411	−0.41805
**Pyrimidine metabolism**				
POPTR_0001s15330.3	Carbamoyl-phosphate synthase small subunit	−1.42227	−0.67993	−0.60156
POPTR_0004s04320.1	DNA-directed RNA polymerase II subunit RPB2	−1.16741	1.003588	1.398599
**Amino Acid Metabolism**				
**Alanine, aspartate and glutamate metabolism**				
POPTR_0006s25830.1	Aspartate aminotransferase, cytoplasmic	−1.42047	2.045731	1.631069
POPTR_0009s07710.1	Asparagine synthase (glutamine-hydrolysing)	−2.14405	3.496922	3.638886
POPTR_0016s13970.1	Alanine-glyoxylate Transaminase/(*R*)-3-amino-2-methylpropionate-pyruvate transaminase	−1.0845	0.637495	0.021722
POPTR_0012s04090.1	Glutamine synthetase	1.656213	−0.20572	−0.19061
POPTR_0015s05010.1	Glutamine synthetase	1.423925	−0.11328	0.999157
POPTR_0008s20460.2	Glutamine synthetase	−1.53133	0.459672	−0.35329
POPTR_0001s15330.3	Carbamoyl-phosphate synthase small subunit	−1.42227	−0.67993	−0.60156
**Glycine, serine and threonine metabolism**				
POPTR_0014s02290.1	D-3-phosphoglycerate dehydrogenase	1.096606	−0.83834	−0.32838
POPTR_0021s00380.1	Aminomethyltransferase	−1.2206	0.226431	0.265644
POPTR_0016s13970.1	Alanine-glyoxylate transaminase/(*R*)-3-amino-2-methylpropionate-pyruvate transaminase	−1.0845	0.637495	0.021722
POPTR_0012s07730.1	Betaine-aldehyde dehydrogenase	−1.90348	−0.18349	−0.54518
POPTR_0006s11980.1	Threonine dehydratase	−2.19346	−0.0663	0.727021

Table S1. *Cont.*

Gene ID	Description	Fold Change (*dwf1*/WT)	Fold Change (XL-5/WT)	Fold Change (XL-6/WT)
**Cysteine and methionine metabolism**				
POPTR_0014s08210.1	L-3-cyanoalanine synthase/cysteine synthase	−1.15984	−0.16325	−0.81118
POPTR_0010s09370.1	Homocysteine S-methyltransferase	−2.68942	0.622164	0.999385
POPTR_0004s10660.1	*S*-adenosylmethionine decarboxylase	−1.90123	−0.63745	−0.63739
POPTR_0017s14280.3	*S*-adenosylmethionine decarboxylase	−1.23619	−0.2682	−0.29005
POPTR_0014s15710.1	Aminocyclopropanecarboxylate oxidase	1.499482	−0.2864	0.155728
POPTR_0006s25830.1	Aspartate aminotransferase, cytoplasmic	−1.42047	2.045731	1.631069
POPTR_0001s11350.1	Methionine-gamma-lyase	−1.22733	2.043488	2.022392
**Valine, leucine and isoleucine degradation**				
POPTR_0012s08010.1	Aldehyde dehydrogenase (NAD^+)	−1.41556	0.479957	−0.21366
**Valine, leucine and isoleucine biosynthesis**				
POPTR_0006s11980.1	Threonine dehydratase	−2.19346	−0.0663	0.727021
POPTR_0003s10720.1	3-Isopropylmalate/(*R*)-2-methylmalate dehydratase small subunit	−1.2432	−0.251	0.057266
POPTR_0004s04370.1	Ketol-acid reductoisomerase	−1.40595	0.000912	0.052826
POPTR_0005s08480.1	2-Isopropylmalate synthase	−1.57022	−0.57116	−0.58386
**Lysine degradation**				
POPTR_0014s15280.1	2-Oxoglutarate dehydrogenase E2 component (dihydrolipoamide Succinyltransferase)	−1.63733	0.076435	0.170827
POPTR_0012s08010.1	Aldehyde dehydrogenase (NAD^+)	−1.41556	0.479957	−0.21366
**Arginine and proline metabolism**				
POPTR_0004s10660.1	*S*-adenosylmethionine decarboxylase	−1.90123	−0.63745	−0.63739
POPTR_0017s14280.3	*S*-adenosylmethionine decarboxylase	−1.23619	−0.2682	−0.29005
POPTR_0012s08010.1	Aldehyde dehydrogenase (NAD^+)	−1.41556	0.479957	−0.21366
POPTR_0006s25830.1	Aspartate aminotransferase, cytoplasmic	−1.42047	2.045731	1.631069
POPTR_0012s04090.1	Glutamine synthetase	1.656213	−0.20572	−0.19061
POPTR_0015s05010.1	Glutamine synthetase	1.423925	−0.11328	0.999157
POPTR_0008s20460.2	Glutamine synthetase	−1.53133	0.459672	−0.35329

Table S1. *Cont.*

Gene ID	Description	Fold Change (*dwf1*/WT)	Fold Change (XL-5/WT)	Fold Change (XL-6/WT)
**Histidine metabolism**				
POPTR_0013s11820.1	Histidinol-phosphate aminotransferase	−1.56169	0.464056	−0.1422
POPTR_0012s08010.1	Aldehyde dehydrogenase (NAD^+)	−1.41556	0.479957	−0.21366
**Tyrosine metabolism**				
POPTR_0006s25830.1	Aspartate aminotransferase, cytoplasmic	−1.42047	2.045731	1.631069
POPTR_0013s11820.1	Histidinol-phosphate aminotransferase	−1.56169	0.464056	−0.1422
POPTR_0002s05840.1	4-Hydroxyphenylpyruvate dioxygenase	−2.37545	0.087065	−0.24602
**Phenylalanine metabolism**				
POPTR_0006s25830.1	Aspartate aminotransferase, cytoplasmic	−1.42047	2.045731	1.631069
POPTR_0013s11820.1	Histidinol-phosphate aminotransferase	−1.56169	0.464056	−0.1422
POPTR_0002s05840.1	4-Hydroxyphenylpyruvate dioxygenase	−2.37545	0.087065	−0.24602
POPTR_0001s04820.1	Peroxidase	1.437159	0.126532	−0.26303
**Tryptophan metabolism**				
POPTR_0014s11910.1	Kynurenine-oxoglutarate transaminase/cysteine-*S*-conjugate β-lyase/glutamine—phenylpyruvate transaminase	−3.04307	1.443919	1.638755
POPTR_0012s08010.1	Aldehyde dehydrogenase (NAD^+)	−1.41556	0.479957	−0.21366
POPTR_0001s34230.1	Cytochrome P450, family 1, subfamily A, polypeptide 1	1.313417	−0.30348	−0.18057
POPTR_0002s02690.1	Cytochrome P450, family 83, subfamily B, polypeptide 1	2.719892	−0.45519	−0.59177
**Phenylalanine, tyrosine and tryptophan biosynthesis**				
POPTR_0005s11300.1	3-Dehydroquinate synthase	−1.30617	0.238396	0.458151
POPTR_0005s04490.1	3-Dehydroquinate dehydratase/shikimate dehydrogenase	1.547488	0.902095	0.877433
POPTR_0008s04040.1	Chorismate synthase	1.745427	−0.16526	0.364078
POPTR_0008s07440.1	Arogenate dehydrogenase ($NADP^+$), plant	−1.88575	0.801127	−0.13797
POPTR_0006s25830.1	Aspartate aminotransferase, cytoplasmic	−1.42047	2.045731	1.631069
POPTR_0013s11820.1	Histidinol-phosphate aminotransferase	−1.56169	0.464056	−0.1422

Table S1. *Cont.*

Gene ID	Description	Fold Change (*dwf1*/WT)	Fold Change (XL-5/WT)	Fold Change (XL-6/WT)
**Glutathione metabolism**				
POPTR_0002s20880.1	Glutathione S-transferase	2.711999	0.371969	0.584963
POPTR_0483s00220.1	Glutathione S-transferase	2.028403	0.683922	0.424733
POPTR_0013s00660.1	Glucose-6-phosphate 1-dehydrogenase	−1.26949	−0.18314	0.374822
POPTR_0001s09270.2	Glutathione peroxidase	−1.35916	−0.05965	0.224187
POPTR_0001s09280.1	Glutathione peroxidase	−1.91269	0.294408	0.219082
POPTR_0009s13650.1	L-ascorbate peroxidase	−1.38204	0.143221	−0.36323
**Metabolism of Cofactors and Vitamins**				
**Porphyrin and chlorophyll metabolism**				
POPTR_0004s14560.1	Ferrochelatase	−1.35201	−0.00687	−0.03942
POPTR_0016s02570.1	Magnesium-protoporphyrin IX monomethyl ester (oxidative) cyclase	1.057457	−0.85298	−0.25922
POPTR_0001s41370.1	Protochlorophyllide reductase	1.416154	0.468741	0.329946
POPTR_0004s22680.1	Pheophorbide a oxygenase	−1.54976	−0.0895	−0.36422
POPTR_0017s07890.1	Glucuronosyltransferase	1.10191	0.043625	−0.20886
POPTR_0008s06270.2	Glucuronosyltransferase	−2.35411	0.088461	0.088461
POPTR_0008s07270.2	Ferritin heavy chain	−1.40998	0.509709	−0.23178
POPTR_0016s13270.3	Ferritin heavy chain	−2.23599	2.993992	2.518902
**Genetic Information Processing**				
**Transcription**				
**RNA polymerase**				
POPTR_0004s04320.1	DNA-directed RNA polymerase II subunit RPB2	−1.16741	1.003588	1.398599
**Spliceosome**				
POPTR_0012s08650.1	ATP-dependent RNA helicase DDX5/DBP2	−3.66297	0.624535	0.394086
POPTR_0006s11790.1	Splicing factor U2AF 35 kDa subunit	−1.23457	0.27542	0.094304
POPTR_0001s06720.1	Peptidyl-prolyl isomerase H (cyclophilin H)	−1.23054	−0.87907	−0.5681
POPTR_0013s01160.2	U4/U6.U5 tri-snRNP component SNU23	−2.06386	−0.35769	−0.77973
POPTR_0013s01350.1	Pre-mRNA-splicing factor 38A	−1.03829	−0.37174	−0.92123

Table S1. *Cont.*

Gene ID	Description	Fold Change (*dwf1*/WT)	Fold Change (XL-5/WT)	Fold Change (XL-6/WT)
**Spliceosome**				
POPTR_0010s21270.1	Heat shock 70 kDa protein 1/8	1.146157	0.455569	−0.32149
POPTR_0010s21350.1	Heat shock 70 kDa protein 1/8	−4.43838	−0.37348	−0.42114
POPTR_0006s03080.1	Polyglutamine-binding protein 1	−1.8771	−0.19858	0.534759
POPTR_0004s06590.1	Coiled-coil domain-containing protein 12	−1.41336	0.423808	−0.5979
POPTR_0005s09680.2	ATP-dependent RNA helicase	1.24075	0.634488	0.925911
POPTR_0013s01700.1	Splicing factor, arginine/serine-rich 1/9	−1.55276	0.398323	0.115725
POPTR_0002s09630.1	Splicing factor, arginine/serine-rich 2	−1.64697	0.065751	−0.25008
**Translation**				
**Ribosome**				
POPTR_0011s02660.1	Small subunit ribosomal protein S14e	−1.46581	−0.84558	−0.66661
POPTR_0010s24760.1	Small subunit ribosomal protein S17e	1.028118	0.400952	0.605187
POPTR_0012s08850.1	Small subunit ribosomal protein S30e	1.312837	0.913405	0.569779
POPTR_0010s02340.1	Large subunit ribosomal protein L14	−1.48242	−0.17921	0.071207
POPTR_0006s13470.1	Large subunit ribosomal protein L4e	−1.06513	−0.50032	−0.59239
POPTR_0014s17230.1	Large subunit ribosomal protein L5e	−1.54998	0.450341	−0.09579
POPTR_0009s07020.1	Large subunit ribosomal protein L6e	−1.45444	0.305583	−0.02975
POPTR_0004s21300.1	Large subunit ribosomal protein L10Ae	−1.14403	−0.61308	−0.53156
POPTR_0006s19530.1	Large subunit ribosomal protein L11e	1.126197	0.796978	0.29567
POPTR_0010s08060.1	Large subunit ribosomal protein L14e	−1.17277	−0.70328	−0.64528
POPTR_0002s20400.1	Large subunit ribosomal protein L18e	−1.81707	−0.6495	−0.16767
POPTR_0011s06730.1	Large subunit ribosomal protein L18Ae	−2.24361	−0.62056	−0.06596
POPTR_0012s03450.1	Large subunit ribosomal protein L19e	−1.65357	0.400519	−0.21005
POPTR_0017s11850.1	Large subunit ribosomal protein L34e	−1.11489	−0.78685	−0.53184
POPTR_0011s07390.1	Large subunit ribosomal protein L36e	−1.69682	0.747278	−0.77968
POPTR_0010s18850.1	Large subunit ribosomal protein L38e	1.459885	0.883066	0.773442

Table S1. *Cont.*

Gene ID	Description	Fold Change (*dwf1*/WT)	Fold Change (XL-5/WT)	Fold Change (XL-6/WT)
**Aminoacyl-tRNA biosynthesis**				
POPTR_0008s17260.1	Aspartyl-tRNA(Asn)/glutamyl-tRNA (Gln) amidotransferase subunit B	−1.55186	−0.83531	−0.44726
POPTR_0014s04810.1	Glycyl-tRNA synthetase	−2.28174	0.070001	−0.2562
POPTR_0010s21430.1	Methionyl-tRNA synthetase	−1.15085	−0.60308	−0.91985
03013 RNA transport	?	?	?	?
POPTR_0006s12140.1	Nuclear pore complex protein Nup205	−1.22352	2.003391	1.217048
POPTR_0004s10350.1	Translation initiation factor eIF-2B subunit delta	−1.3233	0.469594	−0.3336
POPTR_0011s03020.1	Translation initiation factor 4E	−1.43138	−0.88711	−0.2662
POPTR_0005s09680.2	ATP-dependent RNA helicase	1.24075	0.634488	0.925911
**mRNA surveillance pathway**				
POPTR_0005s09680.2	ATP-dependent RNA helicase	1.24075	0.634488	0.925911
POPTR_0013s09190.1	Peptide chain release factor subunit 3	−1.05344	0.001748	−0.22473
**Ribosome biogenesis in eukaryotes**				
POPTR_0003s15120.1	U3 small nucleolar RNA-associated protein 10	−1.67743	−0.18763	−0.50637
POPTR_0001s17340.1	Periodic tryptophan protein 2	−3.04307	−0.33927	−0.69945
POPTR_0012s08430.1	U3 small nucleolar RNA-associated protein MPP10	−1.20209	−0.11048	0.139223
POPTR_0009s12370.1	Nucleolar protein 4	−1.02324	−0.32977	−0.54597
**Protein export**				
POPTR_0018s13680.1	Preprotein translocase subunit YidC	−2.19022	0.016874	−0.05845
POPTR_0009s05750.1	Translocation protein SEC62	1.382605	0.858049	0.931287
**Protein processing in endoplasmic reticulum**				
POPTR_0009s05750.1	Translocation protein SEC62	1.382605	0.858049	0.931287
POPTR_0015s12070.1	Calnexin	1.417766	0.330003	0.127832
POPTR_0018s13790.1	Mannosyl-oligosaccharide α-1,2-mannosidase	1.159674	−0.17457	−0.32149
POPTR_0010s21800.1	Protein transport protein SEC24	−1.15391	−0.25734	−0.53842
POPTR_0008s03990.3	Protein disulfide-isomerase A1	1.293863	0.640149	0.735131
POPTR_0021s01020.1	Derlin-2/3	−1.03713	0.111324	0.340356

Table S1. *Cont.*

Gene ID	Description	Fold Change (*dwf1*/WT)	Fold Change (XL-5/WT)	Fold Change (XL-6/WT)
**Protein processing in endoplasmic reticulum**				
POPTR_0010s21270.1	Heat shock 70 kDa protein 1/8	1.146157	0.455569	−0.32149
POPTR_0010s21350.1	Heat shock 70 kDa protein 1/8	−4.43838	−0.37348	−0.42114
POPTR_0017s01160.1	Molecular chaperone HtpG	#NUM!	−0.3179	−0.30953
POPTR_0006s08830.1	Hsp70-interacting protein	−1.59488	−0.53997	0.161115
POPTR_0009s05450.1	Hsp20 family protein	#NUM!	−0.37817	−0.06598
POPTR_0010s20300.1	Hsp20 family protein	−6.49985	−0.68992	−0.92574
POPTR_0013s09600.1	Hsp20 family protein	#NUM!	−0.91972	−0.16713
**SNARE interactions in vesicular transport**				
POPTR_0014s14210.1	Syntaxin 7	−1.22105	0.418567	−0.0917
04131 SNAREs	?	?	?	?
**Ubiquitin mediated proteolysis**				
POPTR_0013s06060.2	Ubiquitin-conjugating enzyme E2 A	−1.11782	0.433525	0.747015
POPTR_0001s41030.4	E3 ubiquitin-protein ligase RFWD2	1.040647	0.392674	0.264874
POPTR_0014s12890.1	RING finger and CHY zinc finger domain-containing protein 1	−1.95576	−0.26204	−0.78691
POPTR_0002s07540.1	F-box and leucine-rich repeat protein 1 (*S*-phase kinase-associated protein 2)	−1.16441	−0.59229	−0.83071
POPTR_0010s10320.1	Cullin 3	−1.37324	0.146712	−0.08997
POPTR_0011s08280.1	Cullin 4	−1.01444	0.181658	0.393327
POPTR_0014s01640.1	DNA excision repair protein ERCC-8	−1.64182	−0.26582	−0.55223
**Proteasome**				
POPTR_0009s13560.1	26S proteasome regulatory subunit N10	−1.12455	0.041792	0.527287
POPTR_0010s10860.1	26S proteasome regulatory subunit T6	−1.06621	0.638747	−0.94728
**RNA degradation**				
POPTR_0016s05590.1	Exosome complex component RRP43	2.904805	0.979759	0.936073
POPTR_0002s00380.1	Superkiller protein 3	−1.53127	−0.89328	0.455822
POPTR_0011s08710.1	CCR4-NOT transcription complex subunit 9	−1.46875	−0.482	−0.58973
POPTR_0004s23310.1	Molecular chaperone DnaK	−1.6338	−0.37333	−0.42424

Table S1. *Cont.*

Gene ID	Description	Fold Change (*dwf1*/WT)	Fold Change (XL-5/WT)	Fold Change (XL-6/WT)
**Signal Transduction**				
**Two-component system**				
POPTR_0012s04090.1	Glutamine synthetase	1.656213	−0.20572	−0.19061
POPTR_0015s05010.1	Glutamine synthetase	1.423925	−0.11328	0.999157
POPTR_0008s20460.2	Glutamine synthetase	−1.53133	0.459672	−0.35329
**MAPK signaling pathway**				
POPTR_0014s13840.1	Protein phosphatase 5	−1.21402	−0.27304	−0.44359
POPTR_0010s21270.1	Heat shock 70 kDa protein 1/8	1.146157	0.455569	−0.32149
POPTR_0010s21350.1	Heat shock 70 kDa protein 1/8	−4.43838	−0.37348	−0.42114
**Calcium signaling pathway**				
POPTR_0003s09450.1	Calmodulin	1.838009	0.018737	−0.30902
POPTR_0013s01200.1	Calmodulin	1.352315	−0.06879	0.131384
**Phosphatidylinositol signaling system**				
POPTR_0001s02250.1	Inositol-1,3,4-trisphosphate 5/6-kinase/inositol-tetrakisphosphate 1-kinase	1.241355	−0.0779	−0.21579
POPTR_0016s01220.2	Myo-inositol-1(or 4)-monophosphatase	−1.34605	0.382187	0.800661
POPTR_0003s09450.1	Calmodulin	1.838009	0.018737	−0.30902
POPTR_0013s01200.1	Calmodulin	1.352315	−0.06879	0.131384
**Plant hormone signal transduction**				
POPTR_0005s16020.1	Auxin influx carrier (AUX1 LAX family)	1.930864	−0.12063	0.181026
POPTR_0019s13450.1	Auxin responsive GH3 gene family	2.667425	−0.73906	−0.17701
POPTR_0013s14740.1	Auxin responsive GH3 gene family	#NUM!	2.602535	3.141356
POPTR_0009s12890.1	SAUR family protein	2.963163	0.531447	−0.1669
POPTR_0012s03050.1	SAUR family protein	1.161831	−0.09823	0.82939
POPTR_0014s06240.1	SAUR family protein	1.523053	0.676783	−0.07205
POPTR_0015s00920.1	SAUR family protein	1.301033	−0.50919	0.556393
POPTR_0014s13200.1	Histidine−containing phosphotransfer peotein	−1.40483	−0.87907	−0.87712
POPTR_0013s02980.1	Gibberellin receptor GID1	−1.17487	0.115984	−0.66409

Table S1. *Cont.*

Gene ID	Description	Fold Change (*dwf1*/WT)	Fold Change (XL-5/WT)	Fold Change (XL-6/WT)
**Plant hormone signal transduction**				
POPTR_0014s13170.1	Gibberellin receptor GID1	−2.04134	1.24664	1.761213
POPTR_0014s02220.1	F-box protein GID2	1.379387	0.389632	0.910188
POPTR_0010s19120.1	Abscisic acid receptor PYR/PYL family	2.94422	−2.22687	−1.02251
POPTR_0012s01550.1	Abscisic acid receptor PYR/PYL family	−1.58117	−0.21671	0.155795
POPTR_0010s20720.1	Protein phosphatase 2C	−1.35377	0.761897	0.873132
POPTR_0009s04210.1	Protein phosphatase 2C	#NUM!	3.313417	2.823737
POPTR_0009s11010.1	Serine/threonine-protein kinase SRK2	−1.32932	0.458477	−0.05526
POPTR_0002s12710.1	ABA responsive element binding factor	−1.39873	0.697847	0.76797
POPTR_0009s10400.1	ABA responsive element binding factor	−1.81729	−0.26219	−0.56068
POPTR_0008s16430.1	Ethylene receptor	−1.00207	1.649063	1.638464
POPTR_0018s01710.1	EIN3-binding F-box protein	1.224986	0.793968	0.523509
POPTR_0008s06460.1	Coronatine-insensitive protein 1	−1.06575	0.139908	0.357881
POPTR_0001s02450.1	Transcription factor MYC2	1.74573	0.094188	0.545172
